# Data on the removal of metals *(Cr^3+^, Cr^6+^, Cd^2+^, Cu^2+^, Ni^2+^, Zn^2+^)* from aqueous solution by adsorption using magnetite particles from electrochemical synthesis

**DOI:** 10.1016/j.dib.2019.103956

**Published:** 2019-04-27

**Authors:** Manrique-Julio Jorge, Marriaga-Cabrales Nilson, Hernández-Ramírez Aracely, Fiderman Machuca-Martínez

**Affiliations:** a Escuela de Ingeniería Química, Universidad del Valle, A.A. 23360, Cali, Colombia; b Facultad de Ciencias Químicas, Universidad Autónoma de Nuevo León, Ave. Universidad S/N, Cd. Universitaria, San Nicolás de los Garza, N.L. C.P. 64450, Mexico

## Abstract

Magnetic materials are promising adsorbents for removing heavy metals from polluted wastewaters. Magnetite particles were prepared by electrolytic synthesis (average crystallite size 37.9±1.2 nm, surface area = 17.2 ${\text{m}}^{2}{\text{g}}^{-1}$, isoelectric point = 6.3, magnetic saturation = 62 emu g^−1^) and used as adsorbent of heavy metals in aqueous solutions. The adsorption capacity of the magnetite was highly dependent on pH value, for Cd^+2^, Zn^+2^, Ni^+2^ and Cu^+2^ the removal performance was higher that 80% at pH = 8. For Cr+6, the acid pH showed removal percentage higher that 90%.

The adsorbent was separated from the system, reactivated and reused in subsequent tests using batch adsorption. It was found that removal efficiencies were higher than 70% even during a third cycle of adsorption. Finally, the kinetic behavior of the adsorption of each adsorbate was described by a first-order. The range of values of q_e_(mg/g) and k (min^−1^) were 1.3166–1.6367 and 0.0377 to 0.0826 respectively.

**Table:** Specifications table

Subject area	Chemical engineering
More specific subject are	Adsorption process
Type of data	Figures and tables
How data was acquired	The magnetite particles was obtained by electrochemical synthesis. XRD, N_2_ Adsorption, ZPC and magnetic properties was used in the characterization of materials. Data were obtained by adsorption test from cation solutions at room conditions.
Data format	Analyzed
Experimental factors	All experimental tests were performance to laboratory scale.
Experimental features	Metal ions adsorption using magnetite obtained by electrochemical synthesis were investigated.
Data source location	Universidad del Valle, Cali, Colombia and Universidad Autonoma de Nuevo León, Monterrey, México.
Data accessibility Related research article	The data is found only in this article. J. Manrique-Julio, F. MacHuca-Martinez, N. Marriaga-Cabrales, M. Pinzon-Cardenas, Production of magnetite by electrolytic reduction of ferric oxyhydroxide, J. Magn. Magn. Mater. (2016). https://doi.org/10.1016/j.jmmm.2015.10.018

**Table:** 

**Value of data** • A new method to produce magnetite with high specific area was used as heavy metals adsorbent in aqueous solutions • The removal of ${\text{Cr}}^{6+}$, ${\text{Cd}}^{2+}$, ${\text{Cu}}^{2+}$, ${\text{Ni}}^{2+}$ and ${\text{Zn}}^{2+}$ from synthetic wastewater onto magnetite was performed using batch adsorption. • Adsorption tests performed without an adsorbent alkaline pretreatment suggested that the process is primarily driven by electrostatic attraction. • The adsorption kinetics was found to follow a Pseudo-first order model for every metal ion.

## 1 Data

**Fig. 1:**
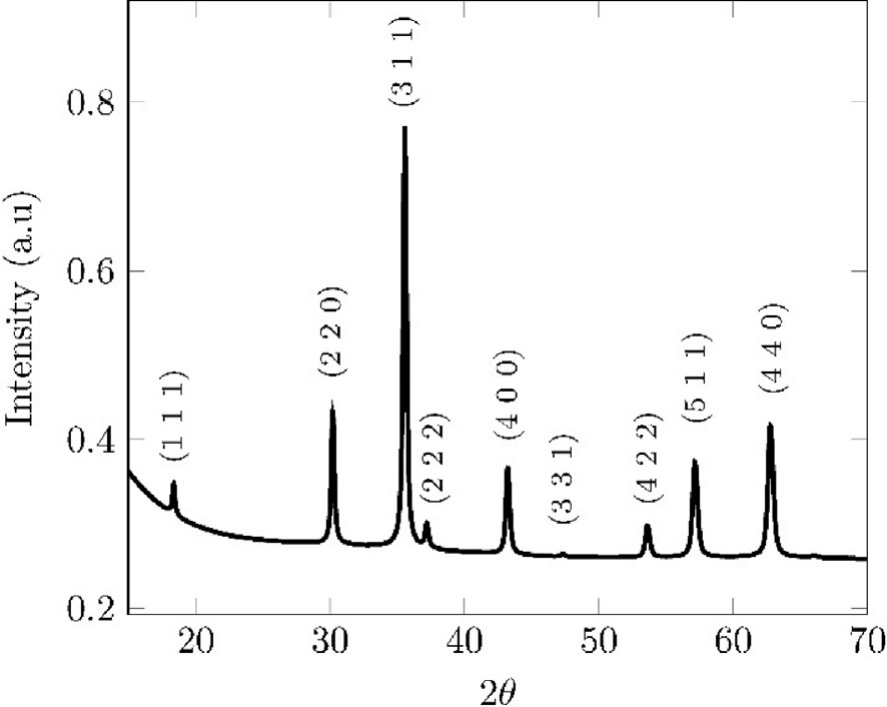
XRD diffractogram of magnetite particles obtained at 18 $mA\;cm^{-2}$ and a distance between electrodes of 0.3 *cm*.

**Fig. 2:**
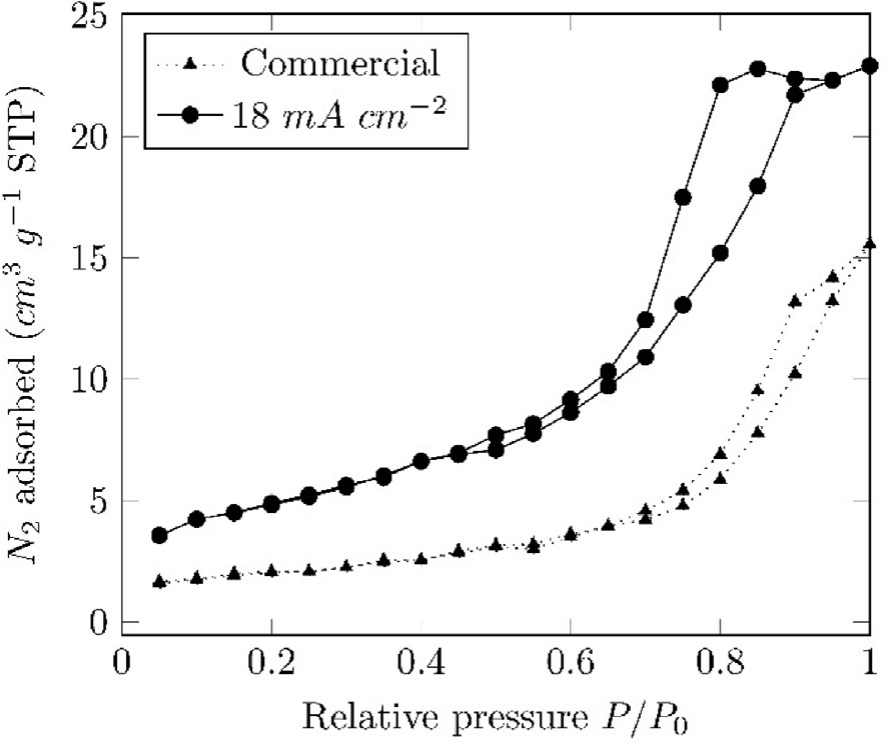
N_2_ adsorption/desorption isotherms of commercial (Sigma-Aldrich) and electrolytically prepared magnetite at 18 $mA\;cm^{-2}$.

**Fig. 3:**
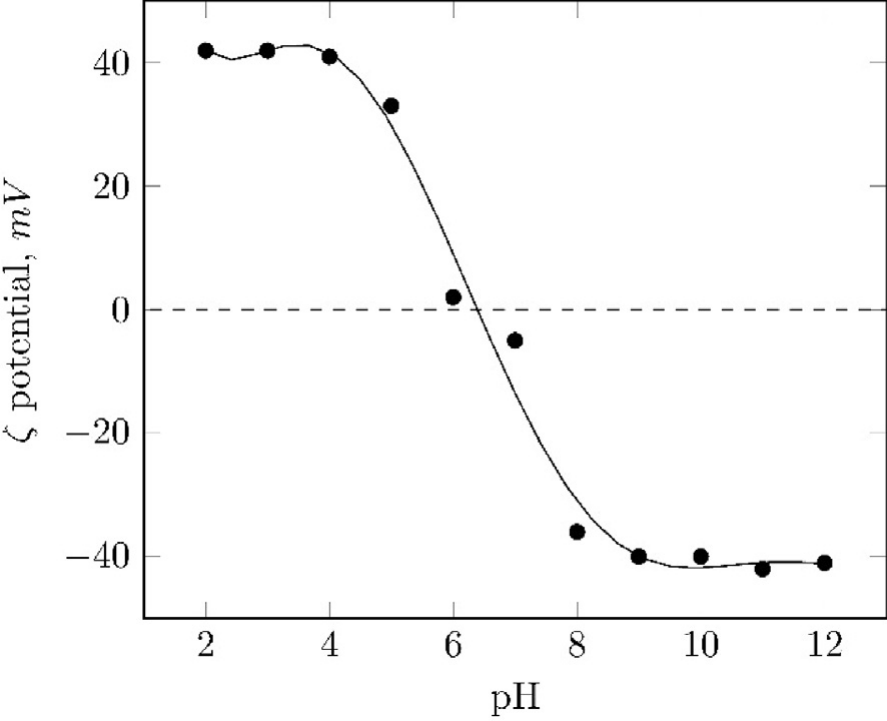
Zeta potential values of the magnetite particles in a NaCl solution, 100$mg\;l^{-1}$.

**Fig. 4:**
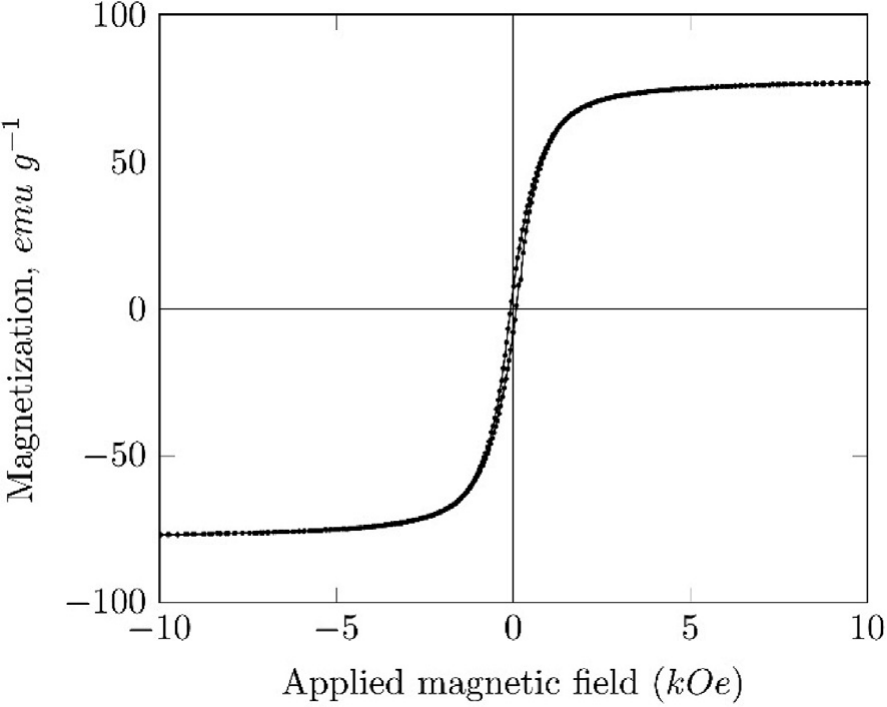
Magnetization curve of magnetite obtained by Vibrating Sample Magnetometry at 300K.

This brief data set describes the electrochemical synthesis of magnetite particles (Fig. 1), the Fig. 2, Fig. 3, Fig. 4 shows the N_2_ isotherms, zeta potential and magnetization hysteresis curves.

**Fig. 5:**
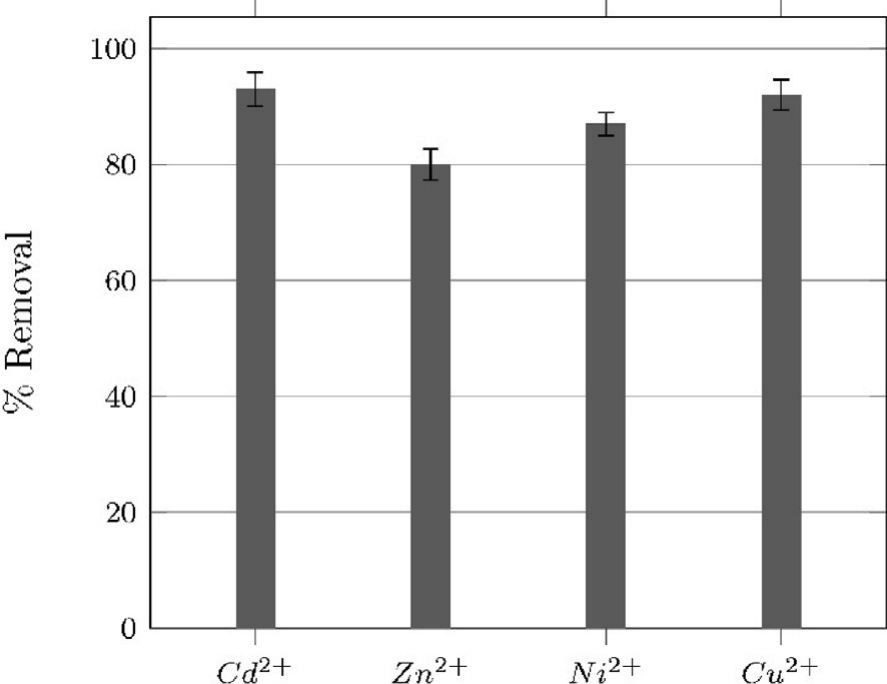
Removal of $Cd^{2+}$, $Zn^{2+}$, $Ni^{2+}$ and $Cu^{2+}$ cations from a 50 $mg\;l^{-1}$ aqueous solutions at pH 8.0 during 120 min.

Fig. 5 shows adsorption capacity of magnetite on metal ions (Cd^+2^, Zn^+2^, Ni^+2^, Cu^+2^) solutions, the Table 1 shows the molar distribution of the ions versus pH.

**Table 1:** Theoretical speciation of the metal cations in aqueous media and their molar distribution.

Cation	Species	Molar distribution (%)
${\text{Cd}}^{2+}$	${\left[\text{Cd}{\left({\text{OH}}_{2}\right)}_{6}\right]}^{2+}$	99
	${\left[\text{Cd}\left(\text{OH}\right){\left({\text{OH}}_{2}\right)}_{5}\right]}^{+}$	1
${\text{Zn}}^{2+}$	${\left[\text{Zn}{\left({\text{OH}}_{2}\right)}_{6}\right]}^{2+}$	84
	${\left[\text{Zn}\left(\text{OH}\right){\left({\text{OH}}_{2}\right)}_{5}\right]}^{+}$	7
	$\left[\text{Zn}{\left(\text{OH}\right)}_{2}{\left({\text{OH}}_{2}\right)}_{4}\right]$	9
${\text{Ni}}^{2+}$	${\left[\text{Ni}{\left({\text{OH}}_{2}\right)}_{6}\right]}^{2+}$	99
	${\left[\text{Ni}\left(\text{OH}\right){\left({\text{OH}}_{2}\right)}_{5}\right]}^{+}$	1
${\text{Cu}}^{2+}$	${\left[\text{Cu}{\left({\text{OH}}_{2}\right)}_{6}\right]}^{2+}$	4
	${\left[\text{Cu}\left(\text{OH}\right){\left({\text{OH}}_{2}\right)}_{5}\right]}^{+}$	11
		2
		13
		70

**Fig. 6:**
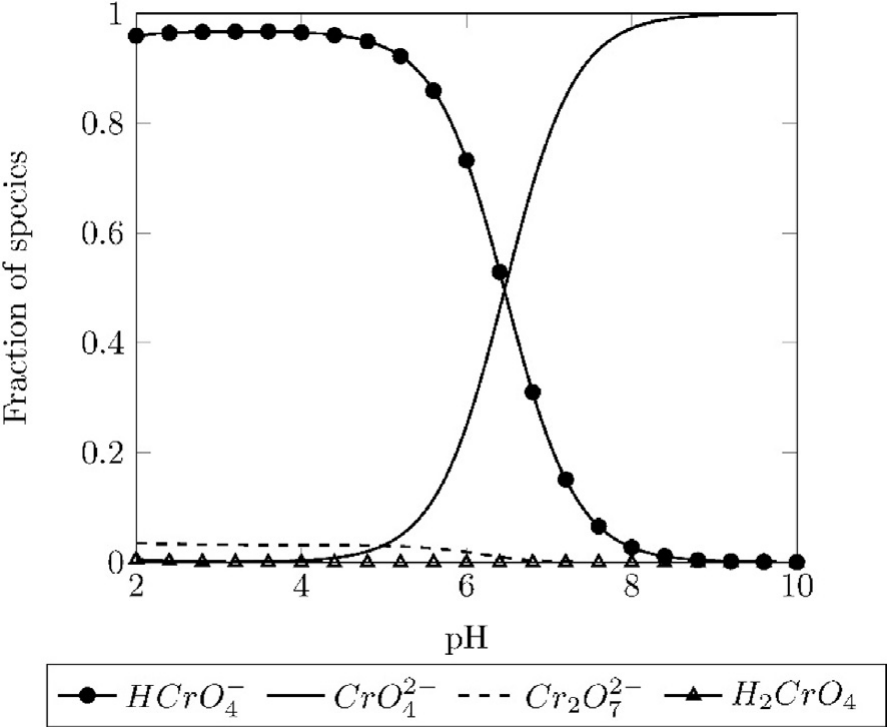
Speciation diagram of hexavalent chromium at concentration of 50 $mg\;l^{-1}$ and room temperature.

**Fig. 7:**
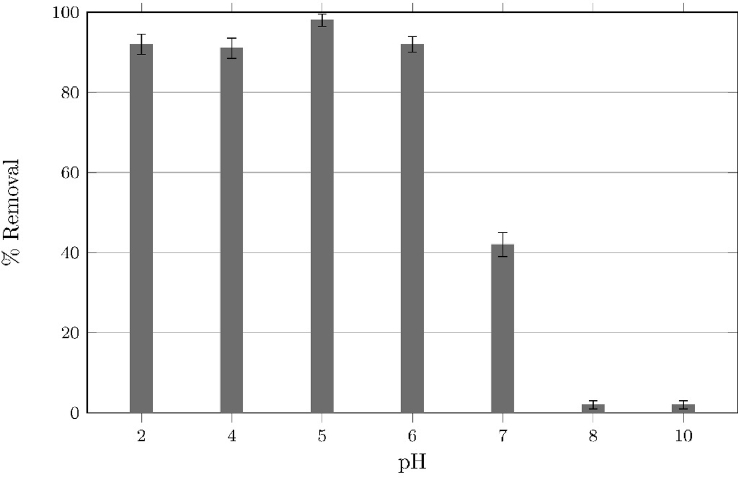
Effect of pH on adsorption efficiency of $Cr^{6+}$ onto magnetite surface, from a 50 $mg\;l^{-1}$ aqueous solutions.

For Cr^+6^, the removal percentage and species type in function of pH are shown in Fig. 6 and Fig. 7 respectively.

**Fig. 8:**
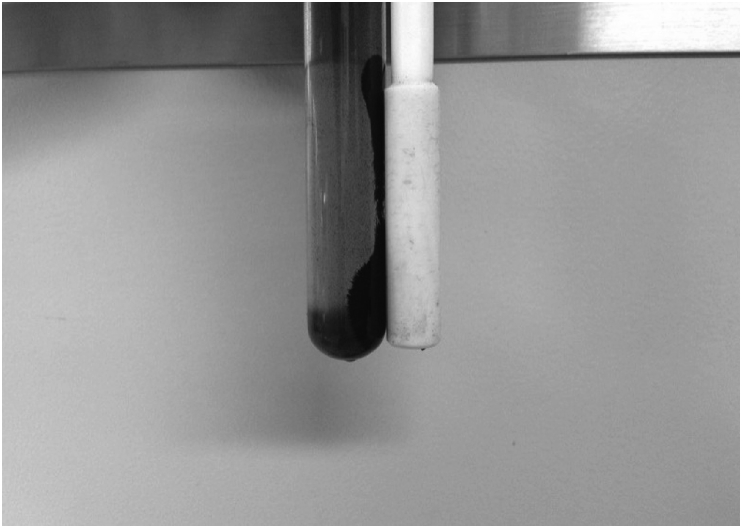
Magnetic separation of the adsorbent particles.

**Fig. 9:**
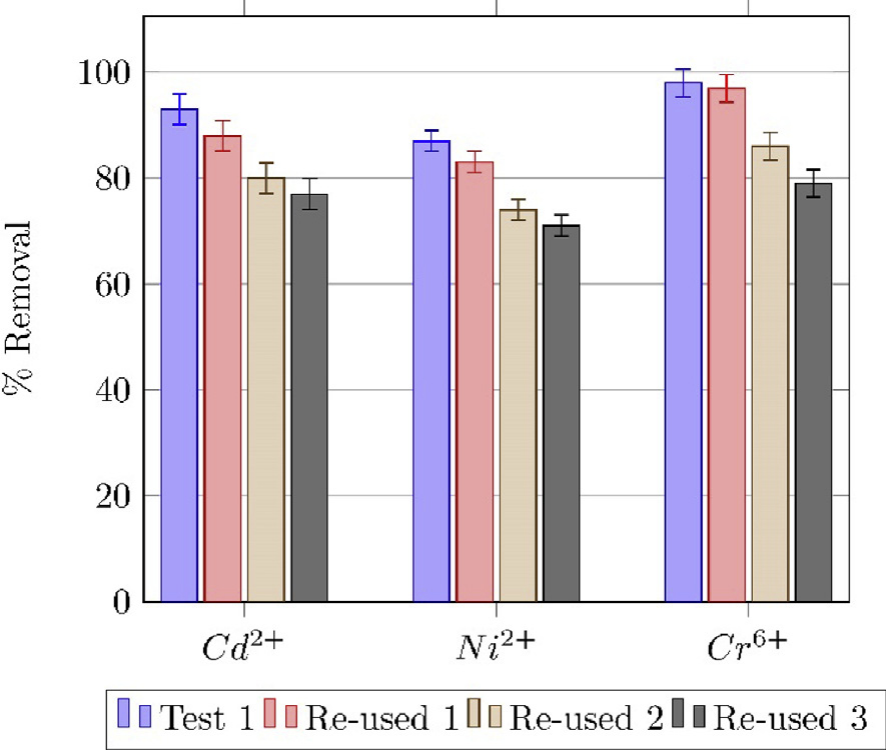
Removal efficiency of metal cations in successive adsorption cycles. $Cd^{2+}$ and $Ni^{2+}$ adsorption pH was 8.0 and 5.0 for$Cr^{6+}$.

The regenerated adsorbent was separated (Fig. 8) and re-used in three adsorption cycles, the Fig. 9 shows the removal percentage for Cd^+2^, Ni^+2^ and Cr^+6^.

**Fig. 10:**
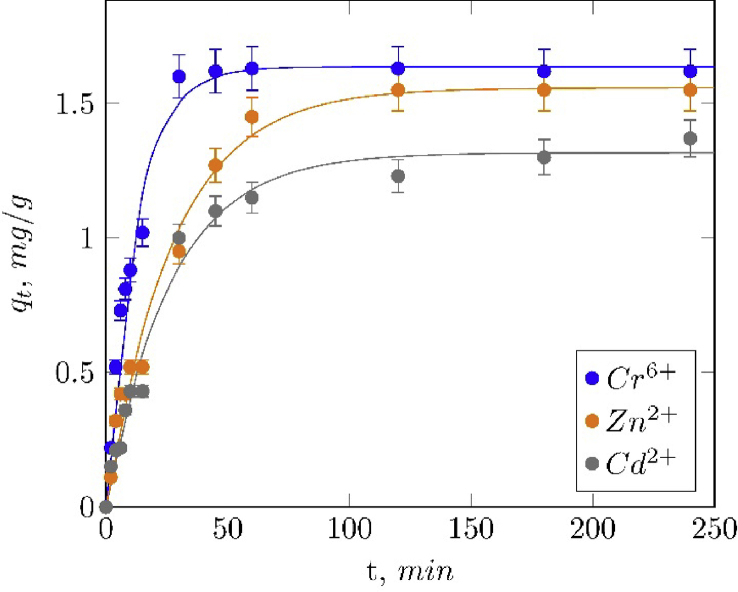
Fitted pseudo-first order adsorption kinetic curves of $Cr^{6+}$, $Zn^{2+}$ and $Cd^{2+}$ in $Fe_{3}O_{4}$.

**Fig. 11:**
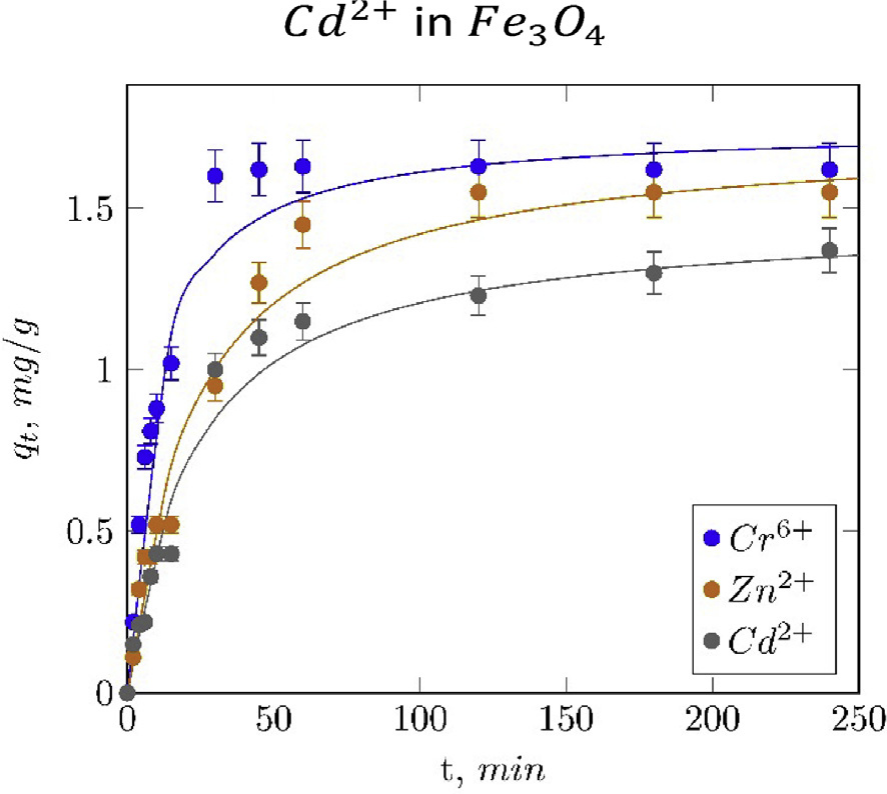
Fitted pseudo-second order adsorption kinetic curves of $Cr^{6+}$, $Zn^{2+}$ and $Cd^{2+}$ in $Fe_{3}O_{4}$.

Finally, the Table 2, Fig. 10 and Fig. 11 shows the adsorption kinetics parameters and kinetics curves of metal ions adsorption on magnetite particles.

**Table 2:** Adsorption kinetic models parameters.

	Pseudo-first order			Pseudo-second order		
	$q_{e}\;\left(\frac{mg}{g}\right)$	$k\;\left(\min^{-1}\right)$	${\text{R}}^{2}$	$q_{e}\;\left(\frac{mg}{g}\right)$	$k\;\left(\min^{-1}\right)$	${\text{R}}^{2}$
${\text{Cr}}^{6+}$	1.6367	0.0826	0.9901	1.7516	0.0659	0.9714
${\text{Cd}}^{2+}$	1.5581	0.0363	0.9883	1.7316	0.0264	0.9786
${\text{Cu}}^{2+}$	1.5302	0.0401	0.9789	1.6912	0.0301	0.9695
${\text{Ni}}^{2+}$	1.4429	0.0444	0.9904	1.5959	0.0345	0.9768
${\text{Zn}}^{2+}$	1.3166	0.0377	0.9879	1.4746	0.0306	0.9769

### 1.1 Magnetite synthesis and characterization

Under the conditions described in the experimental section, following a previously reported methodology, a black precipitate was obtained and identified as pure magnetite (${\text{Fe}}_{3}{\text{O}}_{4}$) by mean of X-Ray diffraction-XRD [1]. Fig. 1 shows the diffractogram of a sample obtained and every peak was identified by comparing with the standard pattern for magnetite (Registry JCPDS number: 01-088-0315) [2].

From Rietveld refinement results (Pseudo-Voigt Model: ${\text{χ}}^{2}<1.5$; $\text{R}\left({\text{F}}^{2}\right)<0.1$), crystalline structure can be classified as Face Centered Cubic (FCC) with space group $\text{Fd}\bar{3}\text{m}$. The crystallite size was calculated in the parallel, equation (1), and perpendicular, equation (2), directions to the growth anisotropy using the Scherrer equations [3]:

(1) $$\phi_{∥}=\frac{18000\;k\;\lambda }{\pi \left(L_{x}+ptec\right)}$$

(2) $$\phi_{⊥}=\frac{18000\;k\;\lambda }{\pi L_{x}}$$

Where k=0.9 is the Scherrer constant, λ = 0.154056 is the wavelength of the ${\text{Cu}\;\text{K}}_{{\text{α}}_{1}}$ radiation, and ${\text{L}}_{\text{x}}$ and ptec are the isotropic and anisotropic Lorentzian broadenings, respectively, of the crystallite size [3]. The ptec value was calculated to be zero for each sample, which indicates that the crystallites were spherical. The average crystallite size of all samples, calculated by Scherrer equation, was found to be 37.9±1.2 nm.

Based on the ${\text{N}}_{2}$ adsorption/desorption isotherm, shown in Fig. 2, The Brunauer, Emmett and Teller - BET surface area of the ${\text{Fe}}_{3}{\text{O}}_{4}$ particles was estimated to be 17.2 ${\text{m}}^{2}{\text{g}}^{-1}$, a value higher than the average specific areas of a commercially available magnetite (6.9 ${\text{m}}^{2}{\text{g}}^{-1}$) and the natural occurring magnetite, < 4 ${\text{m}}^{2}{\text{g}}^{-1}$ [2], [4].

The adsorption/desorption isotherm of the prepared samples can be classified as a Type-IV isotherm with a hysteresis loop in the range of pressure of 0.3–0.95, suggesting that the samples are mesoporous. Pore size, determined by BJH method, was estimated to be around 5 nm, which can be classified as mesoporous (2–50 nm) [2], [6].

The determination of the zeta potential was performed to indicate the sign of the surface charge depending on the pH media and the isoelectric point (IEP). As shown in Fig. 3 the IEP was calculated to be around 6.3.

Below the IEP, the particles developed a positive surface charge owing to a protonation of surface sites and over this value it turned into negative surface charges. Changes in surface charge are due to the protonation and deprotonation of surface hydroxyl groups formed during the pretreatment of the particles with a NaOH solution, equation (3), described by equations (4), (5) for acidic and alkaline media, respectively [5], [7].

(3) $${\left[Fe_{3}O_{4}\right]}_{surf}+OH^{-}⇄{\left[Fe_{3}O_{4}\right]}_{surf}-OH^{-}$$

(4) $${\left[Fe_{3}O_{4}\right]}_{surf}-OH+H^{+}⇄{\left[Fe_{3}O_{4}\right]}_{surf}-OH_{2}^{+}$$

(5) $${\left[Fe_{3}O_{4}\right]}_{surf}-OH+OH^{-}⇄{\left[Fe_{3}O_{4}\right]}_{surf}-O^{-}+H_{2}O$$

Fig. 4 shows the magnetization hysteresis loop of the magnetite particles synthesized. The values of magnetic saturation (${\text{M}}_{\text{s}}$), remanence and coercivity obtained were 62 $\text{emu}\;{\text{g}}^{-1}$, 0.09 kOe and 7.5 $\text{emu}\;{\text{g}}^{-1}$ respectively.

The magnetic saturation obtained from the hysteresis curve was lower than the values reported in the literature, 92–100 $\text{emu}\;{\text{g}}^{-1}$ [2]. This behavior can be attributed to a possible surface oxidation of the magnetite particle into another oxide with a lower ${\text{M}}_{\text{s}}$, like maghemite, ${\text{M}}_{\text{s}}$ = 72 $\text{emu}\;{\text{g}}^{-1}$ [2]. Some authors also attribute this variation of ${\text{M}}_{\text{s}}$ to the synthesis method used to prepare the particles [6], [7].

### 1.2 Heavy metals adsorption tests

The Batch adsorption test for every metal cation at different pH values were performed. Fig. 5 shows the removal percentage obtained for ${\text{Cd}}^{2+}$, ${\text{Zn}}^{2+}$, ${\text{Ni}}^{2+}$ and ${\text{Cu}}^{2+}$ metal cations at pH = 8.

Table 1 shows the theoretical species formed in aqueous media by each metal cation at pH 8.0 and their molar distribution, determined by using Visual Minteq software [8]. At this pH value, the species have a positive formal charge, in contrast to magnetite that shows a negative zeta potential of −36 mV. This difference in charges can explain the high removal efficiencies obtained.

The adsorption tests performed at pH values lower than 8.0 evidenced negligible removal efficiencies (less than 10%). This behavior can be attributed to the fact, that at these conditions, magnetite and the aqueous species formed by the metal cations are positively charged, hindering the attraction between them. According to simulations performed in Visual Minteq, the only species formed by the cations is the hexa-aquo complex, ${\left[\text{M}{\left({\text{OH}}_{2}\right)}_{6}\right]}^{2+}$ (M= ${\text{Cd}}^{2+}$, ${\text{Zn}}^{2+}$, ${\text{Ni}}^{2+}$ or ${\text{Cu}}^{2+}$).

In the adsorption test performed with the Cr(III) solution, removal efficiencies were negligible at pH values less than 7.0. This can be explained by the repelling effect between magnetite (positive surface charge at this pH value) and the species formed at those acidity levels: ${\left[\text{Cr}{\left({\text{OH}}_{2}\right)}_{6}\right]}^{3+}$ and ${\left[\text{Cr}\left(\text{OH}\right){\left({\text{OH}}_{2}\right)}_{5}\right]}^{2+}$. The addition of NaOH to the Cr(III) solution caused the immediate precipitation of the cation in the form of $\left[\text{Cr}{\left(\text{OH}\right)}_{3}{\left({\text{OH}}_{2}\right)}_{3}\right]$ at any alkaline pH level; therefore it was not necessary to perform adsorption test at these conditions [9].

In contrast to trivalent chromium, hexavalent chromium does not precipitate at any pH value [10], i.e. does not produce any quantifiable uncharged aqueous species, Fig. 6. This can be explained by the fact that ${\text{Cr}}^{6+}$ ion can strongly polarize water molecules due to its high formal charge.

Hexavalent chromium adsorption efficiencies in function of pH are shown in Fig. 7. Acidic pH values showed the highest removal values; this can be attributed to the difference in electrostatic charge between magnetite (positive zeta potential) and the species formed in aqueous media by hexavalent chromium (${\text{HCrO}}_{4}^{-}$) shown in the speciation diagram, Fig. 6.

This species, also described as ${\left[{\text{CrO}}_{3}\left(\text{OH}\right)\right]}^{-}$, undergoes a dimerization reaction, equation (6), turning into dichromate ${\text{Cr}}_{2}{\text{O}}_{7}^{2-}$ that can also be attracted by the positive surface charge of magnetite [11].

(6) $$2HCrO_{4}^{-}⇌Cr_{2}O_{7}^{2-}+H_{2}O$$

Low removal values at high pH values can be explained by a similar argument; magnetite and hexavalent chromium repeal each other because of their charges, negative zeta potential and ${\text{CrO}}_{4}^{2-}$ respectively.

The alkaline pretreatment (NaOH solution 1.0 M during 15 minutes prior to each adsorption test) to the adsorbent particles, to increase the number of hydroxyl groups, plays an important role in the adsorption efficiency [11]. Adsorption tests performed without this pretreatment showed low removal percentages (less than 10%) for all the cations; this result suggests that the adsorption could be primarily due to electrostatic attraction between the aqueous cationic species and the charged hydroxyl groups formed onto the surface of magnetite.

### 1.3 Desorption and reuse of the adsorbent

Once the batch adsorption tests finished, magnetite was separated from the solution by using a magnetic retriever, as shown in Fig. 8. The used adsorbent was washed with a 0.05 M NaOH solution of pH 12 and rinsed with distilled water for several times, in order to use it subsequent adsorption tests.

The recovery of the adsorbent was calculated using equation (7).

(7) $$\text{Recovery}=100\frac{N_{Rec}}{N_{Ads}}$$

Where ${\text{N}}_{\text{Ads}}$ and ${\text{N}}_{\text{Rec}}$ are the adsorbed and recovered moles of the metal cation, respectively. The recovery of the adsorbent was between 60 and 80%; this suggests that some of the metal cations could be chemically adsorbed on the surface of magnetite [12], [13].

The regenerated adsorbent was used in subsequent metal cations adsorption tests, and removal efficiencies were as high as 70% even in the third cycle of adsorption, Fig. 9. The decreasing removal capacity of the adsorbent, after each regeneration cycle, suggests that some of the species could be chemically adsorbed onto magnetite surface, as previously reported [14].

#### 1.3.1 Adsorption kinetics

In order to understand the kinetic behavior of the adsorption process, two kinetic models were fitted to the experimental data: pseudo-first and pseudo-second order kinetic models, equations (8), (9), respectively:

(8) $$q_{t}=q_{e}\left(1-e^{-kt}\right)$$

(9) $$q_{t}=\;\;\;\;\frac{kq_{e}^{2}t}{1+kq_{e}t}$$

Where ${\text{q}}_{\text{t}}$ and ${\text{q}}_{\text{e}}$ are the amount of absorbed cations at time t (min) and equilibrium, respectively; and k is the rate constant (${\text{min}}^{-1}$). Best fit estimates of these parameters obtained by a non-linear regression analysis are summarized in Table 2 and the fitting plots using both models are illustrated in Figs. 10 and 11, jointly with the experimental data.

Hexavalent chromium presented the higher values of kinetic constants, evidenced in a fast adsorption rate at the initial stage. This behavior could be attributed to the higher electrostatic attraction between this cation and the adsorption sites of magnetite, unlike the other cations with low formal charge, and to the initial concentration gradient [13], [14], [15].

Pseudo-first order model showed the higher correlations (represented as ${\text{R}}^{2}$), suggesting that this kinetic model is more suitable for describing the adsorption behavior of these metallic cations on magnetite than the pseudo-second order model. This result does not agree with that reported by Martinez et al [14] and Hosseinzadeh et al [13]; in these studies, it was concluded that the pseudo-second order equation fits better, this result could be attributed to the fact that nanosized magnetite was used. The discrepancy in the results obtained could be explained by the fact that in both studies nanosized magnetite was used as adsorbent, able to strongly chemically interact with the cationic species due to size effects.

## 2 Experimental section, materials and methods

### 2.1 Magnetite particles synthesis and characterization

**Fig. 12:**
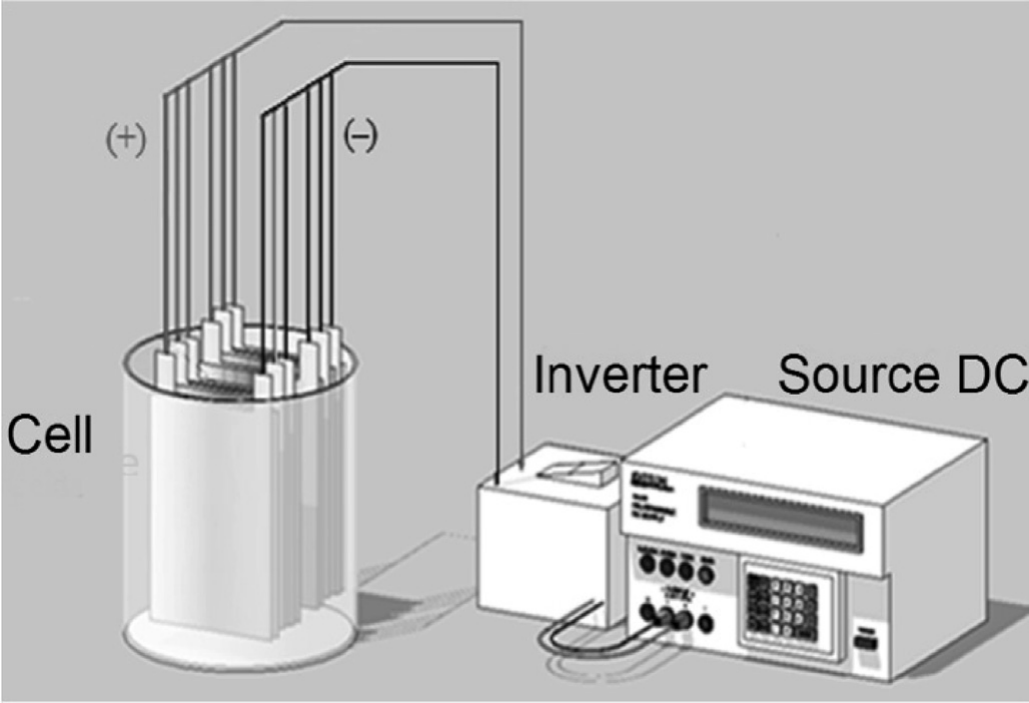
Monopolar electrolytic cell.

Magnetite particles (${\text{Fe}}_{3}{\text{O}}_{4}$) were synthesized by using the electrochemical method using a monopolar arrangement of carbon steel electrodes, immersed in a 0.04 M NaCl solution at neutral pH, and a current density of 18 ${\text{mA}\;\text{cm}}^{-2}$ using an EXTECH 382280 DC power source, up to an electric charge of 3000 CL^−1^ (Fig. 12). The distance between the electrodes was fixed to 0.3 cm and room conditions were used in all experiments, as previously reported [1].

Phase composition and crystallite size of the particles were studied by X-ray diffraction using an X'Pert Pro PANalytical diffractometer (PANalytical, Netherlands). Copper radiation (${\text{λ}}_{\text{Kα}1}$= 1.54056 Å, ${\text{λ}}_{\text{Kα}2}$ = 1.54439 Å) was used in a 2θ range of 15–90° at a step size of 1° at room temperature. The crystallographic analysis was performed by Rietveld method using the X'Pert High Score Plus software (PANalytical, Netherlands).

Surface area and pore size distribution were determined by measuring the ${\text{N}}_{2}$ adsorption isotherms in an ASAP 2020 system (Micromeritics, USA). Previous to each measurement, the samples were degassed at 353 K during 12 h, in order to eliminate physically adsorbed moisture. BET (Brunauer-Emmet-Teller) and BJH (Barret-Joyner-Halenda) methods were used for determining the specific surface area and pore size, respectively [16], [17].

Point of zero charge of the magnetite was determined in a Zeta-Meter Z402 (Zeta-Meter Inc, USA) by measuring the electrophoretic mobility (μ) of the particles in a 100 ppm NaCl solution in a pH range of 2–12, adjusted by adding drops of HCl and NaOH. From the obtained electrophoretic mobility, zeta potential (ζ) was calculated using the Smoluchowski, equation (10):

(10) $$\text{ζ}=\;\frac{4\;\pi \eta }{\varepsilon }\mu$$

Where η is the viscosity of the suspension medium and ε the viscosity of the fluid phase.

Magnetic properties measurement of the samples was carried out using a PPMS (Physical Properties Measurement System, Quantum Design, San Diego - USA) in VSM mode (Vibrating Sample Magnetometry) with a sweeping from −10 to +10 kOe at 300 K.

### 2.2 Preparation of heavy metal solutions

All heavy metal solutions, with a concentration of 50 $\text{mg}\cdot {\text{l}}^{-1}$, were prepared by dilution, with ultrapure water of type 1, of standard solutions (Atomic absorption standard solutions with a concentration of 1000 $\text{mg}\cdot {\text{l}}^{-1}$). Table 3 summarizes the information concerning the standard solutions used.

**Table 3:** Standard solutions used for preparing the aqueous solutions of heavy metals.

Cation	Standard solution
${\text{Cd}}^{2+}$	SC118-500 Fisher
${\text{Zn}}^{2+}$	SZ13-500 Fisher
${\text{Ni}}^{2+}$	1092 Karal
${\text{Cu}}^{2+}$	SC194 Fisher
${\text{Cr}}^{3+}$	EW-86995-42 Ricca
${\text{Cr}}^{6+}$	ACR61KW-500 Ricca

### 2.3 Adsorption tests

Adsorption tests of metal cations (${\text{Cd}}^{2+}$, ${\text{Zn}}^{2+}$, ${\text{Ni}}^{2+}$, ${\text{Cu}}^{2+}$, ${\text{Cr}}^{3+}$ and ${\text{Cr}}^{6+}$) were performed in batch mode, using 250 ml of each solution, over a wide range of pH (2–10) at room conditions. pH values were adjusted by adding drops of HCl (0.5 M) or NaOH (0.5 M) for acidic or alkaline conditions, respectively.

Adsorbent concentration was fixed to a previously determined value, 30 $\text{g}\cdot {\text{l}}^{-1}$, and the particles were treated with a NaOH solution (1.0 M) for 15 minutes, prior to each adsorption test, to increase the number of hydroxyl groups onto the surface of the particles [12]. A reciprocating shaker bath (Thermo Scientific 2870, Massachusetts) was used to agitate the solution/adsorbent mixture at 120 rpm for 120 minutes.

After contact time, adsorbent particles were removed from solution by using a magnetic retriever and treated again with a NaOH solution with a concentration of 1.0 M to desorb the metal cations and reuse the adsorbent.

The concentration of the metal cations, remaining in solution, was measured by atomic absorption spectroscopy, using a Varian SpectrAA 220 FS spectrometer (Varian Inc, Palo Alto). Metal cation adsorption efficiency was reported as removal percentage (%Rem) and calculated by using equation (11):

(11) $$\text{\%}Rem\;=\;100\frac{C_{i}\;-C_{f}\;\;}{C_{i}}$$

Where ${\text{C}}_{\text{i}}$ and ${\text{C}}_{\text{f}}$ are the initial and final metal cations concentrations respectively.
