# Supplementary material for: Data on the removal of metals (Cr3+, Cr6+, Cd2+, Cu2+, Ni2+, Zn2+) from aqueous solution by adsorption using magnetite particles from electrochemical synthesis
Source: Data Brief. 2019 Apr 27;24:103956. doi: 10.1016/j.dib.2019.103956 (PMC6517579; doi:10.1016/j.dib.2019.103956)
Supplement: Multimedia component 1 [file mmc1.docx]

Santiago de Cali, 6/02/2019

Data in Brief

# Editors

Dear Editors

The authors of the paper entitled: *DATA ON THE REMOVAL OF METALS (Cr^3+^, Cr^6+^, Cd^2+^, Cu^2+^, Ni^2+^, Zn^2+^) FROM AQUEOUS SOLUTION BY ADSORPTION USING MAGNETITE PARTICLES FROM ELECTROCHEMICAL SYNTHESIS* by Jorge Manrique-Juli, Nilson Marriaga-Cabrales, Aracelly Hernández-Ramírez, Fiderman Machuca Martínez do not have conflicts of interest for the publication of this work.

Yours sincerely

Prof. Fiderman Machuca Martinez

Corresponding author
